# Correction: Malizia et al. Response of a Radiology Department to the SARS-CoV-2 Pandemic: The Experience of the Hospital “Policlinico Tor Vergata” in Rome. *Int. J. Environ. Res. Public Health* 2021, *18*, 5255

**DOI:** 10.3390/ijerph19084688

**Published:** 2022-04-13

**Authors:** Andrea Malizia, Laura Filograna, Francesco Paolo Sbordone, Giorgio Ciccarese, Andrea Carbone, Beatrice Carreri, Colleen Patricia Ryan, Gian Marco Ludovici, Andrea Chierici, Guglielmo Manenti

**Affiliations:** 1 Department of Biomedicine and Prevention, University of Rome Tor Vergata, Via di Montpellier 1, 00133 Rome, Italy; malizia@ing.uniroma2.it (A.M.); manenti@med.uniroma2.it (G.M.); 2 Policlinico Tor Vergata: Fondazione PTV, Via di Montpellier 1, 00133 Rome, Italy; laura.filograna@ptvonline.it (L.F.); francescopaolo.sbordone@students.uniroma2.eu (F.P.S.); giorgiociccarese@gmail.com (G.C.); andrea.carbone.20@students.uniroma2.eu (A.C.); beatrice.carreri@students.uniroma2.eu (B.C.); c.ryan@hsantalucia.it (C.P.R.); 3 Department of Industrial Engineering, University of Rome Tor Vergata, Via del Politecnico 1, 00133 Rome, Italy; a.chierici@studenti.unipi.it; 4 School of Engineering, University of Pisa Largo Lucio Lazzarino, 2, 56122 Pisa, Italy

All the figures [1] have been removed in the new version of the paper.

- Figure 1: This image refers to the University. We cannot publish an image of the University talking about the Hospital (even if the Hospital is a University Hospital, the image was the entrance of the Didactic Building), so it has been removed.
- Figure 2: This image is of a room from the Department of Nuclear Medicine, and being a paper on the Department of Radiology, we had to remove it.
- Figure 3: This image represents the PPE used before the pandemic situation; the actual PPE is different, so the image has to be removed.

We wish to remove the figures because they are not necessary for the overall significance of the paper. The authors apologize for any inconvenience caused and state that the scientific conclusions are unaffected. The original article has been updated.
